# Classification of PR-positive and PR-negative subtypes in ER-positive and HER2-negative breast cancers based on pathway scores

**DOI:** 10.1186/s12874-021-01297-8

**Published:** 2021-05-22

**Authors:** Taobo Hu, Yan Chen, Yiqiang Liu, Danhua Zhang, Jiankang Pan, Mengping Long

**Affiliations:** 1 Department of Breast Disease, Peking University People’s Hospital, Beijing, China; 2 College of Life Sciences, University of Chinese Academy of Sciences, Beijing, China; 3 Department of Pathology, Peking University Cancer Hospital, Beijing, China; 4 Department of General Surgery, The Second Xiangya Hospital, Central South University, Changsha, Hunan China; 5 Department of Orthopedics, The Second Xiangya Hospital, Central South University, Changsha, Hunan China

**Keywords:** Estrogen receptor, Progesterone receptor, Pathway activities, LASSO, Growth factor

## Abstract

**Purpose:**

PR loss in ER+/HER2- breast cancer indicates worse prognosis and insensitivity to anti-estrogen therapy, while the mechanisms of PR loss in ER+/HER2- breast cancer remain unrevealed.

**Methods:**

In this study, ER+/PR+/HER2- and ER+/PR-/HER2- breast cancer cases from TCGA were used. 1387 pathways were analyzed and used as variables for classifying the two groups with LASSO regression.

**Results:**

ER+/PR+/HER2- and ER+/PR-/HER2- breast cancer groups can be classified by a combination of 13 pathways using their activity score. Among the 13 pathways, those involving growth factors and ion-channel transporters were most significant in the distinction, followed by pathways involving immune modulation and cell metabolism. Two growth factor pathways, EGF and IGF-1, were deferentially regulated in ER+/PR+/HER2- and ER+/PR-/HER2- groups.

**Conclusions:**

In conclusion, this study indicated in ER+/HER2- breast cancers the various status of PR expression can be an indication of molecular variation, particularly for the growth factor pathway activation.

**Supplementary Information:**

The online version contains supplementary material available at 10.1186/s12874-021-01297-8.

## Introduction

About 80 % of breast cancers were hormonal receptor-positive which means they express at least one of the two hormonal receptors, estrogen receptor (ER) and progesterone receptor (PR) [1, 2]. Both ER and PR are ligand-activated transcription factors that promote the expression of specific gene sets by binding to their promoters [3]. Although the expression of ER and PR were often closely correlated and highly consistent, there is still discordance in some breast cancers. It was reported that 15 % of ER-positive breast cancer were PR negative while in PR-positive breast cancer, only 2 % were ER-negative [4], which suggests that ER expressed more widely than PR. Indeed, about 12 % of all breast cancer patients have the hormonal receptor status as ER+/PR- [4]. One of the possible mechanisms for PR loss could be the copy number loss of the *PGR* gene which encodes for PR.

The expression of PR was mostly controlled by activated ER [5], while it can also be regulated by growth factor pathways [6] and cyclin D1 [7]. Analysis of both the Surveillance, Epidemiology and End Results (SEER) [4] and the National Cancer Database (NCD) [8] datasets has confirmed that ER+/PR- breast cancer has worse survival than ER+/PR + breast cancers. Moreover, it was shown that the ER+/PR- group was more resistant to selective estrogen receptor modulator (SERM) therapies [9, 10]. The exact mechanism of the association between PR loss and worse prognosis was remained to be elucidated although several clues existed [11]. One possible mechanism was that the relative overexpression of HER2 in the ER+/PR- group compared with the ER+/PR + group made ER+/PR- breast cancer resistant to tamoxifen [12, 13]. However, in ER+/HER2- breast cancer, the effect of PR expression on prognosis still exists, indicating the presence of other mechanisms [14]. PR has long been considered as the downstream gene of ER since its expression was shown to be induced by estradiol [15], while recent researches proved that PR has strong negative regulatory effect on ER activity [16]. The complex correlation between ER and PR makes it essential to elucidate the molecular characteristiscs and clinical significance of PR loss in ER+/HER2- breast cancer [16].

In this study, we analyzed and compared the pathway activities in ER+/PR+/HER2- and ER+/PR-/HER2- breast cancer using transcriptomic and gene amplification data from TCGA. The two groups were abbreviated as ER+/PR + and ER+/PR-, thus all the studied patients were HER2- unless otherwise specified.

## Results

### Clinicopathological characteristics and survival analysis of the ER+/PR + and ER+/PR- breast cancer patients

In the TCGA cohort, 592 patients have information on ER and PR expression based on immunohistochemical staining. The expression of ER and PR were reported as the percentage of cells with positive expression. ER + and PR + were defined as expressions of more than 1 %, according to the ASCO/CAP guideline [17].

**Fig. 1:**
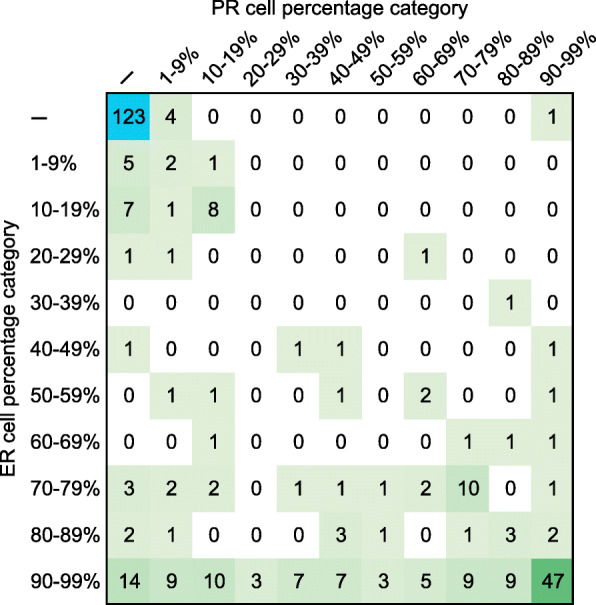
Cell percentage categories of ER and PR expression in HER2- breast cancers from TCGA. The case number of each specific ER and PR expression status was summarized and presented. ER and PR expression were reported either as negative or as the percentage of cells showed positive expression which was subgrouped into 10 categories with a 10 % interval

Cases with positive expression of ER and PR were further subgrouped into 10 categories with a 10 % interval. By this definition, 123 patients belong to the ER-/PR-, while 60 cases were ER+/PR- and 409 cases were ER+/PR+ (Fig. 1).

Clinicopathological characteristics of the ER+/PR- and ER+/PR + groups were compared (Table 1). In concordance with previous studies, the two groups showed a significant difference in clinicopathological characteristics including clinical stage and nodal status. Specifically, ER+/PR- group patients have both more advanced clinical stage and nodal status. In terms of PAM50 intrinsic subtypes distribution of the two groups, it was found that the PR- group was more enriched in luminal B and basal-like type than the PR + group. In addition, comprehensive molecular portraits of the two groups were analyzed including DNA methylation, copy number variation, and miRNA profile which have all been tested and clustered in the previous study [18]. Distributions in methylation cluster and copy number cluster were found to be different between the two groups while no difference was detected in the miRNA cluster distribution. For the DNA methylation cluster, the PR- group was enriched in cluster 5 which was a cluster that overlapped with basal-like mRNA subtype while the PR + group was more enriched in cluster 2 which seems to be a mixture of mRNA subtypes. For copy number cluster, PR- group was enriched in cluster 2 with PR + enriched in cluster 1. Copy number clusters 1 and 2 were previously found to be correlated with luminal A and basal-like subtypes respectively. All the above results indicated that molecularly PR- group was more similar with basal-like subtype and has a more advanced clinical stage than the PR + group.

**Table 1:** Patient Clinicopathological features of the two selected groups and the multivariate and univariate analysis in the TCGA dataset

Characteristics	ER+/PR-/HER2-	ER+/PR+/HER2-	*p*
Age			
< 35	2 (3.3)	11 (2.7)	0.418
35–49	10 (16.7)	97 (23.7)	
50–69	31 (51.7)	218 (53.3)	
70+	17 (28.3)	83 (20.3)	
AJCC Stage^a^			0.005
Stage I	9 (15.0)	83 (20.9)	
Stage II	27 (45.0)	222 (55.8)	
Stage III	19 (31.7)	86 (21.6)	
Stage IV	5 (8.3)	7 (1.8)	
Histology			0.667
Infiltrating Ductal Carcinoma	46 (76.7)	316 (77.3)	
Infiltrating Lobular Carcinoma	6 (10.0)	54 (13.2)	
Mixed Histology	4 (6.7)	16 (3.9)	
Others	4 (6.7)	23 (5.6)	
PAM50			< 0.001
Luminal A	12 (27.9)	181 (65.8)	
Luminal B	18 (41.9)	81 (29.5)	
Basal-like	7 (16.3)	3 (1.1)	
HER2-enriched	4 (9.3)	6 (2.2)	
Normal-like	2 (4.7)	4 (1.5)	
Methylation Cluster^b^			< 0.001
Cluster 1	8 (13.3)	80 (19.6)	
Cluster 2	9 (15.0)	124 (30.3)	
Cluster 3	9 (15.0)	40 (9.8)	
Cluster 4	19 (31.7)	149 (36.4)	
Cluster 5	12 (20.0)	16 (3.9)	
NA	3 (5.0)	0 (0.0)	
CN Cluster^b^			0.001
Cluster 1	17 (30.4)	42 (10.4)	
Cluster 2	13 (23.2)	161 (40.0)	
Cluster 3	11 (19.6)	73 (18.2)	
Cluster 4	9 (16.1)	71 (17.7)	
Cluster 5	6 (10.7)	55 (13.7)	
miRNA Cluster^b^			0.788
Cluster 1	4 (7.8)	29 (7.8)	
Cluster 2	8 (15.7)	70 (18.8)	
Cluster 3	4 (7.8)	18 (4.8)	
Cluster 4	13 (25.5)	107 (28.7)	
Cluster 5	7 (13.7)	33 (8.8)	
Cluster 6	9 (17.6)	83 (22.3)	
Cluster 7	6 (11.8)	33 (8.8)	

^a^*AJCC* The American Joint Committee on Cancer

^b^ The clusters were defined previously by the Cancer Genome Atlas Network [18]

**Fig. 2:**
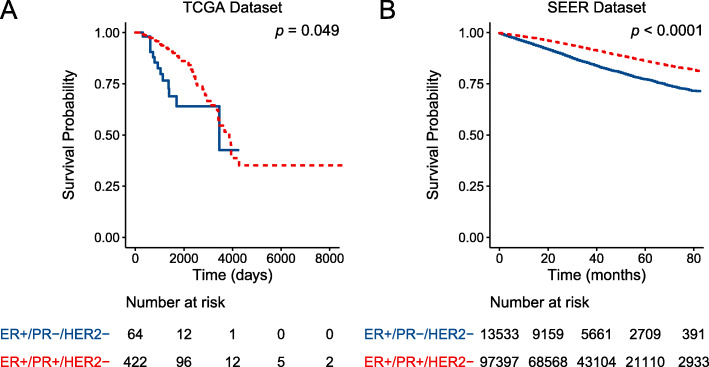
Survival analysis of the ER+/PR + and ER+/PR- groups. Kaplan-Meier survival curves were plotted and the log-rank test was performed to compare the overall survival of ER+/PR + and ER+/PR- groups in TCGA **a** and SEER **b** database

The survival analyses were performed by comparing the overall survival of the ER+/PR-/HER2- and ER+/PR+/HER2- group (Fig. 2). In accordance with previous studies, the overall survival of ER+/PR-/HER2- was significantly poorer than the ER+/PR+/HER2- group. To further validate the result, the same analysis was performed in the Surveillance, Epidemiology, and End Results (SEER) database (Fig. 2). A total of 110,930 ER+/HER2- breast cancer patients were included in the SEER dataset analysis with 97,397 of them being PR + and 13,533 of them being PR-. The clinicopathological characteristics of the included population were presented in Table S1. Li et al. have previously studied the ER+/PR + and ER+/PR- group in the SEER dataset while not considering the status of the HER2 receptor [4].

Both multivariate and univariate analyses were conducted to determine the prognostic value of different clinicopathological features including PR status in all the selected ER+/HER2- patients (Table 2). In univariate analysis, it was found that age, disease stage, tumor size and nodal status were significant prognostic factors for overall survival while factors including age, disease stage, tumor size and copy number cluster were statistically significant in multivariate analysis.

**Table 2:** Univariate and multivariate analysis of the prognostic value of clinicopathological features in ER+/HER2- patients using Cox proportional hazard modeling in the TCGA dataset

Dependent	No.	HR (univariable)	HR (multivariable)
Subtype			
ER+/PR-/HER2-	60	-	-
ER+/PR+/HER2-	409	0.65 (0.34–1.25, *p =* 0.196)	0.77 (0.21–2.84, *p =* 0.697)
Age			
70+	100	-	-
50–69	249	0.60 (0.34–1.05, *p =* 0.075)	0.21 (0.08–0.57, *p =* 0.002)
35–49	107	0.26 (0.12–0.57, *p =* 0.001)	0.05 (0.01–0.22, *p* < 0.001)
< 35	13	1.05 (0.31–3.57, *p =* 0.941)	0.00 (0.00-Inf, *p =* 0.999)
AJCC Stage			
Stage I	92	-	-
Stage II	249	1.45 (0.66–3.18, *p =* 0.354)	8.22 (1.55–43.47, *p =* 0.013)
Stage III	105	1.75 (0.74–4.15, *p =* 0.200)	6.55 (0.47–92.01, *p =* 0.163)
Stage IV	12	7.58 (2.90-19.79, *p* < 0.001)	133.80 (4.74-3775.01, *p =* 0.004)
Histology			
IDC	362	-	-
ILC	60	0.69 (0.29–1.61, *p =* 0.387)	1.35 (0.32–5.71, *p =* 0.682)
Mixed Histology	20	0.82 (0.29–2.27, *p =* 0.700)	2.06 (0.39–10.89, *p =* 0.396)
Others	27	1.29 (0.55–3.04, *p =* 0.562)	1.14 (0.16–7.96, *p =* 0.893)
pT			
T1	138	-	-
T2	256	1.30 (0.70–2.38, *p =* 0.406)	0.27 (0.07–1.03, *p =* 0.055)
T3	54	0.92 (0.38–2.18, *p =* 0.844)	0.05 (0.01–0.39, *p =* 0.005)
T4	20	3.41 (1.42–8.23, *p =* 0.006)	0.08 (0.01–1.05, *p =* 0.055)
pN			
N0	219	-	-
N1	157	1.58 (0.89–2.82, *p =* 0.117)	1.13 (0.32–3.92, *p =* 0.850)
N2	61	2.14 (1.03–4.45, *p =* 0.042)	3.42 (0.38–30.62, *p =* 0.271)
N3	24	2.20 (0.75–6.44, *p =* 0.149)	0.36 (0.04–3.52, *p =* 0.382)
PAM50			
Luminal A	193	-	-
Luminal B	99	1.89 (1.02–3.51, *p =* 0.044)	3.09 (0.97–9.85, *p =* 0.057)
Basal-like	10	0.62 (0.15–2.61, *p =* 0.511)	8.21 (0.95–71.15, *p =* 0.056)
HER2-enriched	10	3.83 (1.14–12.84, *p =* 0.030)	20.69 (3.20-133.85, *p =* 0.001)
Normal-like	6	0.00 (0.00-Inf, *p =* 0.997)	0.00 (0.00-Inf, *p =* 0.999)
Methylation Cluster			
Cluster 1	88	-	-
Cluster 2	133	1.55 (0.75–3.19, *p =* 0.239)	0.89 (0.28–2.88, *p =* 0.852)
Cluster 3	49	0.85 (0.27–2.67, *p =* 0.780)	0.34 (0.05–2.48, *p =* 0.287)
Cluster 4	168	1.70 (0.84–3.44, *p =* 0.142)	0.87 (0.29–2.65, *p =* 0.813)
Cluster 5	28	0.22 (0.03–1.71, *p =* 0.148)	0.00 (0.00-Inf, *p =* 0.997)
CN Cluster			
Cluster 1	59	-	-
Cluster 2	174	0.73 (0.36–1.48, *p =* 0.381)	0.26 (0.08–0.90, *p =* 0.033)
Cluster 3	84	0.50 (0.20–1.21, *p =* 0.124)	0.07 (0.01–0.35, *p =* 0.001)
Cluster 4	80	0.72 (0.33–1.58, *p =* 0.410)	0.07 (0.01–0.40, *p =* 0.003)
Cluster 5	61	0.90 (0.38–2.14, *p =* 0.810)	0.24 (0.05–1.17, *p =* 0.077)
miRNA Cluster			
Cluster 1	33	-	-
Cluster 2	78	0.62 (0.22–1.73, *p =* 0.363)	0.22 (0.03–1.56, *p =* 0.130)
Cluster 3	22	0.40 (0.08–1.92, *p =* 0.250)	0.03 (0.00-0.72, *p =* 0.030)
Cluster 4	120	0.63 (0.26–1.53, *p =* 0.308)	0.39 (0.10–1.63, *p =* 0.200)
Cluster 5	40	1.00 (0.36–2.77, *p =* 1.000)	1.96 (0.33–11.82, *p =* 0.461)
Cluster 6	92	0.64 (0.26–1.56, *p =* 0.330)	0.58 (0.11–3.06, *p =* 0.524)
Cluster 7	39	0.55 (0.17–1.74, *p =* 0.308)	0.38 (0.04–3.66, *p =* 0.403)

### Classification of ER+/PR + and ER+/PR- breast cancer with pathway activities using LASSO methodology

**Fig. 3:**
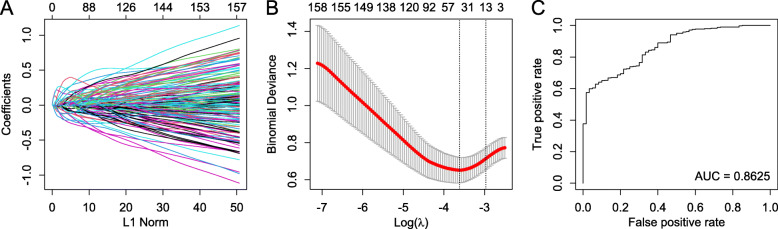
Classifying ER+/PR + and ER+/PR- using 1387 pathways with LASSO methodology **a** Plots for LASSO regression coefficients over different values of the penalty parameter. **b** Cross-validation plot for the penalty term. **c** Receiver operating characteristic curve of the final model

A strategy named least absolute shrinkage and selection operator (LASSO) was used to select the most accurate and compact set of pathways that can differentiate the ER+/PR + and ER+/PR- group. Logistic-LASSO is a regression method that minimizes the usual sum of squared errors which penalizes the regression coefficients. From the 1387 variables/pathways pool, LASSO was able to reduce the number of pathways needed down to thirteen in the final model (Fig. 3). The final model with all the selected pathways and statistics is shown in Table 3. In the final model, the ER+/PR+/HER2- and ER+/PR-/HER2- groups can be distinguished by pathway activities of the 13 pathways, achieving a 0.8625 Area Under the receiver operation characteristic Curve (AUC) (Fig. 3). The most distinct pathways between PR + and PR- groups were growth factors and ion-channel transporter. Also, the FOXA1 pathway which represents the luminal features, and the NOTCH1 pathway which controls cell proliferation were included in the selected pathways.

**Table 3:** Pathways selected in the final model by LASSO

Feature	Estimate
(Intercept)	1.27457
Class_C/3_(Metabotropic_glutamate/pheromone_receptors)	-0.03500
FOXA1_transcription_factor_network	0.10619
Signaling_by_Type_1_Insulin-like_Growth_Factor_1_Receptor_(IGF1R)	0.21581
Amino_acid_transport_across_the_plasma_membrane	-0.01470
Sema3A_PAK_dependent_Axon_repulsion	-0.07956
Na_/Cl-_dependent_neurotransmitter_transporters	0.02917
Metal_ion_SLC_transporters	0.05884
IGF-1_signaling_pathway	0.09220
Signaling_by_constitutively_active_EGFR	-0.04876
Reduction_of_cytosolic_Ca___levels	-0.13606
NOTCH1_Intracellular_Domain_Regulates_Transcription	-0.04873
Synthesis_of_Leukotrienes_(LT)_and_Eoxins_(EX)	0.06633
Activation_of_C3_and_C5	0.23174

### EGFR and IGF-1 pathway were deferentially regulated in ER+/PR + and ER+/PR- breast cancer

**Fig. 4:**
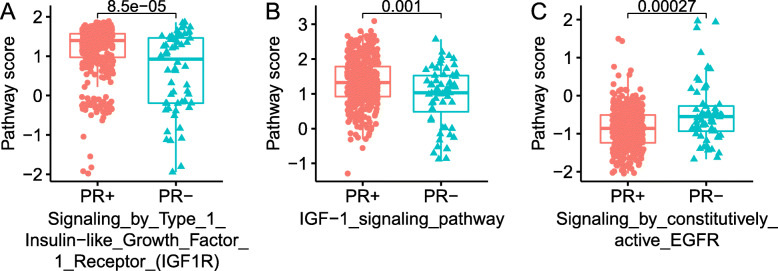
Pathway scores of EGFR and IGF-1 pathways between the PR + and PR- groups. Box plot of the pathway scores in three selected pathways by LASSO between the two groups

In the 13 selected pathways used in the final model, three of them were associated with growth factors including epidermal growth factor receptor (EGFR) and insulin-like growth factor-1 (IGF-1), indicating the direct association between growth factor pathway activation and PR loss. Previous studies have shown that in breast cancer cell lines, activation of EGF or IGF-1 pathways can sharply lower the expression of PR [6]. However, in this study, it was found that the EGFR pathway was more activated in the PR- group while the IGF-1 pathway was more activated in the PR + group (Fig. 4).

**Fig. 5:**
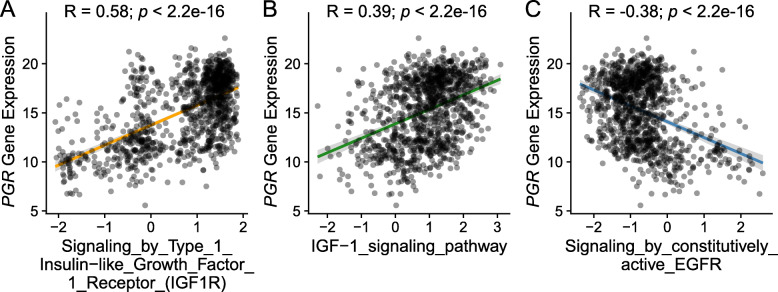
Correlation between growth factor pathway activity with PR expression. Correlation plot of the pathway activity in EGFR and IGF-1 pathways with the mRNA expression value of *PGR* gene in all the samples. Regression line, correlation co-efficiency and *p*-value were also displayed

**Fig. 6:**
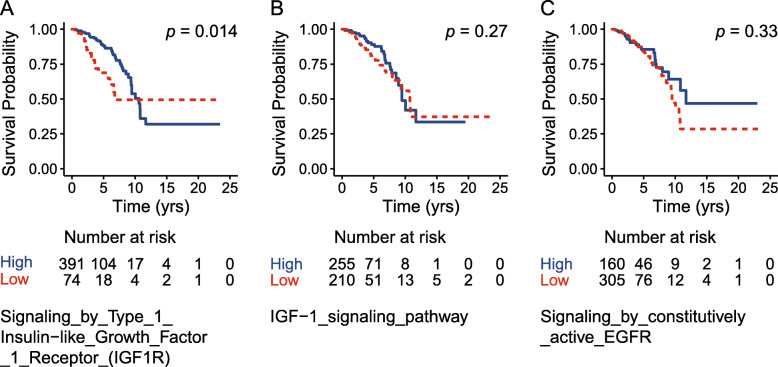
Survival analysis of the pathway activity defined subgroups. Kaplan-Meier survival curves were plotted and the log-rank test was performed to compare the overall survival of low and high activity subgroups as defined by the pathway activity of the IGF1R **a**, IGF-1 **b** and EGFR **c** pathway respectively

The correlation between the pathway score with the mRNA expression value of the *PGR* gene was plotted (Fig. 5). The IGF-1 pathway was found to be positively correlated with the expression of *PGR* while the EGFR pathway was negatively correlated, consistent with our above findings. To further look at the impact of the three growth factor pathways on the prognosis, survival analysis was performed between the pathway-defined subtypes by classifying the studied TCGA population into high-activity and low-activity groups according to the value of pathway score (Fig. 6). In the three analyzed pathways, only the IGF1R pathway-defined two groups showed survival difference while the other two pathways did not show a significant difference which was not surprising due to the fact that the IGF1R pathway score showed the greatest difference between PR + and PR- group in Fig. 4. The insignificance of prognosis between the IGF-1 and EGFR defined groups could possibly be explained by the complexity of the PR loss mechanism which was contributed by a network of pathways.

## Discussion

Although PR loss in ER+/HER2- breast cancer had a worse prognosis and showed insensitivity towards SERM therapy, the molecular mechanism related to PR loss remains to be equivocal. In this study, we analyzed ER+/PR + and ER+/PR- breast cancer in the TCGA cohort. The PR- group was found to be more enriched in basal-like mRNA subtype than the PR + group. Also, in terms of DNA methylation and copy number alteration features, the PR- group was also more similar to the basal-like subtype than the PR + group. Pathway activities which are calculated from transcriptomic and gene copy number data were comprehensively analyzed and compared between the two groups. The PR + and PR- groups could be classified using the pathway activities of 13 selected pathways. Growth factor pathways including EGFR and IGF-1 were found to be essential for the distinction of the two groups which agrees with previous studies. Although previous studies showed that both EGFR and IGF-1 pathway could suppress the PR expression in a way independent of ER [6, 19], our results found that while IGF-1 pathway activity was negatively correlated with the expression of PR, the EGFR pathway activity showed the opposite. Expression of PR was previously found to be repressed by activation of the IGF-1 pathway through PI3K/Akt/mTOR signaling which was independent of ER [6]. Also, genes in PI3K/Akt/mTOR signaling pathways were upregulated in ER+/PR- breast cancer patients compared with ER+/PR + patients [20]. For the EGF pathway, studies were showing that activation of EGFR by adding EGF could enhance the phosphorylation of PR and promote its transcription activity [21], which could explain the positive correlation identified in our result between the EGFR pathway and PR expression. Other important pathways contributing to the classifying were ion-channel transporting pathway, immune-modulating pathway, and cell metabolism pathway. The above findings suggest that in ER+/HER2- breast cancers the various status of PR expression can be an indication of molecular variation, particularly for the growth factor pathway activation. Although, current clinical practice treats ER+/PR+/HER2- and ER+/PR-/HER2- breast cancer patients in the same way, our study suggests that the PR- group should be more intensively studied to search for an effective therapy due to its poor prognosis. Our study, together with other researches, suggested that growth factor pathways such as IGF-1 pathway could be served as potential targets.

## Methods

### Data acquisition and pre-processing

UCSC Xena, an online exploration tool for public and private, multi-omic, and clinical/phenotype data, was used to download data of selected samples [22]. The ‘TCGA Breast Cancer (BRCA)’ cohort in the UCSC Xena was selected. All raw data used to generate Fig. 1; Table 1 was downloaded from the ‘Phenotypes TCGA Hub’, pathway scores were downloaded from the ‘z score of 1387 constituent PARADIGM pathways TCGA Hub’. The pathway scores were generated by the TCGA [23], using the PARADIGM-inferred activation of pathway features [24]. Surveillance, Epidemiology, and End Results (SEER) 18 registries research database (Nov 2018 Submission) was used for the analysis which includes cases diagnosed from 1975 to 2016 [25].

### Feature selection and LASSO regression

Regularized regression is often applied in genetic studies of molecular phenotypes to select the most promising set of variants associated with a phenotype of interest [26]. A widely applied regularized regression method is LASSO, which adds a penalty term for the shrinkage of the parameter estimates to the least-squares loss function [27].

Feature selection and LASSO regression were performed using the “glmnet” R package [28]. The ‘cv.glmnet’ function was used to do the k-fold cross-validation for glmnet, and the lambda.1se of the result was used as the optimal lambda. The glmnet function was used to fit a generalized linear model via penalized maximum likelihood. The alpha penalty was set to be 1 and the family was set to be ‘binomial’ in the glmnet function. The ‘coef.glmnet’ function was used to extract coefficients from the object generated by the glmnet function, with the ‘s’ argument being the optimal lambda generated by the cv.glmnet function.

### Statistical analysis

Survival analyses were performed using the ‘survival’ (version 2.41) package [14]. The Kaplan-Meier method was used to estimate the survival outcomes of all patients by different categories; groups were compared using the log-rank statistic [29]. For univariate and multivariate analysis, the prognostic values of different clinicopathological factors were calculated by Cox proportional hazard modeling. The optimum threshold for distinguishing low and high activity groups was in similar method as described in our previous study [30]. *P* values were calculated as two-sided, with statistical significance declared for *P* less than 0.05.

## Supplementary Information

Additional file 1. **Table S1.** Clinicopathological features of the two selected groups in the SEER dataset. **Table S2.** Univariate and multivariate analysis of the prognostic value of clinicopathological features in ER+/HER2- patients in the SEER dataset.

## Data Availability

The dataset supporting the conclusions of this article is included within the manuscript.

## Abbreviations

AUC: Area Under the receiver operation characteristic Curve.
EGFR: Epidermal Growth Factor Receptor.
ER: Estrogen Receptor.
HER2: Human Epidermal growth factor Receptor 2.
IDC: Infiltrating Ductal Carcinoma.
IGF-1: Insulin-like Growth Factor-1.
ILC: Infiltrating Lobular Carcinoma.
LASSO: Least Absolute Shrinkage and Selection Operator.
NCD: National Cancer Database.
PR: progesterone receptor.
SEER: Surveillance, Epidemiology, and End Results.
SERM: Selective Estrogen Receptor Modulator.
TCGA: The Cancer Genome Atlas.
