# Supplementary material for: Classification of PR-positive and PR-negative subtypes in ER-positive and HER2-negative breast cancers based on pathway scores
Source: BMC Med Res Methodol. 2021 May 22;21:108. doi: 10.1186/s12874-021-01297-8 (PMC8141178; doi:10.1186/s12874-021-01297-8)
Supplement: Supplementary file 1 — Table S1. Clinicopathological features of the two selected groups in the SEER dataset. Table S2. Univariate and multivariate analysis of the prognostic value of clinicopathological features in ER+/HER2- patients in the SEER dataset. [file 12874_2021_1297_MOESM1_ESM.docx]

Table S1. Clinicopathological features of the two selected groups in the SEER dataset

| Characteristics | ER+/PR-/HER2- | ER+/PR+/HER2- | p |
| --- | --- | --- | --- |
| Age |  |  |  |
| <35 | 262 (1.9) | 1123 (1.2) | <0.001 |
| 35-49 | 1487 (11.0) | 16417 (16.9) |  |
| 50-69 | 7186 (53.3) | 49438 (50.9) |  |
| 70+ | 4558 (33.8) | 30187 (31.1) |  |
| Stage |  |  |  |
| Stage 0 | 149 (1.3) | 1880 (2.3) | <0.001 |
| Stage I | 5215 (47.0) | 43954 (54.4) |  |
| Stage II | 2668 (24.0) | 17653 (21.8) |  |
| Stage IIIA | 1830 (16.5) | 11811 (14.6) |  |
| Stage IIIB | 579 (5.2) | 2655 (3.3) |  |
| Stage IV | 656 (5.9) | 2897 (3.6) |  |
| Differentiation Grade |  |  |  |
| Well | 2886 (21.4) | 31321 (32.2) | <0.001 |
| Moderately | 5384 (39.9) | 47879 (49.3) |  |
| Poorly | 4493 (33.3) | 14546 (15.0) |  |
| Undifferentiated | 33 (0.2) | 69 (0.1) |  |
| Unknown | 697 (5.2) | 3351 (3.4) |  |
| Ethnicity |  |  |  |
| Asian | 1211 (9.0) | 9481 (9.8) | <0.001 |
| Black | 1809 (13.4) | 8177 (8.4) |  |
| Other | 183 (1.4) | 1560 (1.6) |  |
| White | 10290 (76.3) | 77948 (80.2) |  |
| Surgery |  |  |  |
| No | 1379 (10.2) | 6716 (6.9) | <0.001 |
| Lumpectomy | 6978 (51.7) | 55937 (57.6) |  |
| Mastectomy | 5136 (38.1) | 34513 (35.5) |  |
| Chemotherapy |  |  |  |
| No | 7971 (59.1) | 72703 (74.8) | <0.001 |
| Yes | 5522 (40.9) | 24463 (25.2) |  |
| Radiation therapy |  |  |  |
| No | 6577 (48.7) | 43217 (44.5) | <0.001 |
| Yes | 6916 (51.3) | 53949 (55.5) |  |

Table S2. Univariate and multivariate analysis of the prognostic value of clinicopathological features in ER+/HER2- patients in the SEER dataset

| Dependent | No. | HR (univariable) | HR (multivariable) |
| --- | --- | --- | --- |
| PR status |  |  |  |
| Negative | 13493 | - | - |
| Positive | 97166 | 1.03 (1.01-1.05, p=0.003) | 1.03 (1.01-1.05, p=0.011) |
| Age |  |  |  |
| <35 | 1385 | - | - |
| 35-49 | 17904 | 0.95 (0.90-1.01, p=0.107) | 0.95 (0.89-1.01, p=0.131) |
| 50-69 | 56624 | 1.00 (0.95-1.06, p=0.980) | 0.97 (0.91-1.03, p=0.337) |
| 70+ | 34745 | 0.99 (0.93-1.04, p=0.610) | 0.93 (0.87-0.99, p=0.025) |
| Stage |  |  |  |
| Stage 0 | 2029 | - | - |
| Stage I | 49169 | 1.06 (1.01-1.10, p=0.020) | 1.06 (1.02-1.11, p=0.008) |
| Stage II | 20321 | 1.04 (0.99-1.09, p=0.100) | 1.08 (1.03-1.13, p=0.002) |
| Stage IIIA | 13641 | 1.02 (0.97-1.07, p=0.534) | 1.09 (1.04-1.15, p=0.001) |
| Stage IIIB | 3234 | 0.96 (0.91-1.02, p=0.231) | 1.04 (0.97-1.10, p=0.263) |
| Stage IV | 3553 | 0.98 (0.91-1.04, p=0.468) | 0.90 (0.83-0.97, p=0.004) |
| Differentiation Grade |  |  |  |
| Well | 34207 | - | - |
| Moderately | 53263 | 0.99 (0.97-1.00, p=0.041) | 0.99 (0.98-1.01, p=0.467) |
| Poorly | 19039 | 0.94 (0.93-0.96, p<0.001) | 0.98 (0.96-1.01, p=0.157) |
| Undifferentiated | 102 | 0.69 (0.56-0.86, p=0.001) | 0.78 (0.62-0.98, p=0.033) |
| Unknown | 4048 | 0.93 (0.90-0.97, p<0.001) | 0.96 (0.92-1.01, p=0.090) |
| Ethnicity |  |  |  |
| Asian | 10692 | - | - |
| Black | 9986 | 0.97 (0.94-1.00, p=0.028) | 0.97 (0.94-1.00, p=0.078) |
| Other | 1743 | 1.08 (1.02-1.14, p=0.005) | 1.06 (1.00-1.13, p=0.043) |
| White | 88238 | 0.93 (0.92-0.95, p<0.001) | 0.93 (0.91-0.96, p<0.001) |
| Surgery |  |  |  |
| No | 8095 | - | - |
| Lumpectomy | 62915 | 0.90 (0.88-0.93, p<0.001) | 0.82 (0.78-0.86, p<0.001) |
| Mastectomy | 39649 | 0.84 (0.82-0.87, p<0.001) | 0.78 (0.75-0.82, p<0.001) |
| Chemotherapy |  |  |  |
| No | 80674 | - | - |
| Yes | 29985 | 0.91 (0.90-0.93, p<0.001) | 0.92 (0.90-0.93, p<0.001) |
| Radiation therapy |  | - | - |
| No | 49794 | - | - |
| Yes | 60865 | 1.01 (0.99-1.02, p=0.361) | 0.99 (0.97-1.01, p=0.209) |
